# Association Between Diabetic Retinopathy and Insomnia Risk: A Nationwide Population-Based Study

**DOI:** 10.3389/fendo.2022.939251

**Published:** 2022-07-14

**Authors:** Yoo Hyun Um, Tae-Won Kim, Jong-Hyun Jeong, Seung-Chul Hong, Ho-Jun Seo, Kyung-Do Han

**Affiliations:** ^1^ Department of Psychiatry, St. Vincent’s Hospital, College of Medicine, The Catholic University of Korea, Seoul, South Korea; ^2^ Department of Statistics and Actuarial Science, Soongsil University, Seoul, South Korea

**Keywords:** retinopathy, diabetes mellitus, insomnia, risk, hazard ratio

## Abstract

**Background:**

Previous studies have suggested a close link between sleep disturbances and diabetic retinopathy (DR). However, to date, no confirmatory findings have been reported. We aimed to explore the risk of insomnia in DR by considering demographic factors and diabetes mellitus (DM)-related variables.

**Methods:**

A nationwide population-based cohort of 2,206,619 patients with type 2 diabetes from the Korean National Insurance Service Database was followed up for insomnia incidence. DR, non-proliferative DR (NPDR), and proliferative DR (PDR) were defined according to ICD-10 codes. The interactive effects of sex, age, and DM-related variables were analyzed to evaluate their impact on insomnia risk in DR.

**Results:**

Compared with the non-DR group, insomnia risk was increased in the DR [(adjusted hazard ratio (aHR): 1.125, 95% confidence interval (CI):1.108-1.142), NPDR (aHR:1.117, 95% CI:1.099-1.134), and PDR (aHR:1.205, 95% CI: 1.156-1.256), even after controlling for comorbidities, lifestyle factors, and DM-related variables. The men and youngest age groups (<40 years) were most vulnerable to insomnia risk. Sex, age, DM duration, and chronic kidney disease (CKD) status exerted interactive effects with DR status in increasing the insomnia risk. In the PDR group, sex, age, DM duration, insulin therapy status, and CKD status exerted interactive effects that increased the risk of insomnia.

**Conclusion:**

Insomnia risk is significantly higher in patients with DR, and clinical attention is warranted.

## Introduction

Diabetic retinopathy (DR) remains a major microvascular complication of diabetes mellitus (DM), with an estimated global prevalence of 22.27%, and the number of affected patients is estimated to increase to 160.5 million by 2045 (1). It has become a leading cause of blindness, and patients with DR frequently report poor quality of life and life satisfaction (2). Moreover, DR severity and visual loss are closely related to negative psychosocial consequences (3).

Regarding the psychiatric consequences of DR, patients are more prone to depression or depressive symptoms (3). In a recent study on patients with chronic eye disease, anxiety levels significantly interacted with visual acuity in determining the quality of life (4). The bidirectional relationship between 1) insomnia and anxiety and 2) insomnia and depression has been frequently reported (5). The retina plays a critical role in transmitting light information to the brain and regulating the human sleep-wake circadian rhythm. Retinal dysregulation can disrupt normal daily rhythms and cause sleep disruption (6). However, despite this crucial relationship, reports on the incidence of insomnia and sleep characteristics in patients with DR are scarce.

Emerging evidence has demonstrated that DR and sleep disturbance are closely related. Recent cross-sectional studies have shown that short or long sleep duration is associated with DR (7, 8). Another study elucidated the positive correlation of sleep quality with DR severity as well as increased sleep latency in patients with DR (9). Among elderly subjects with diabetes, DR was more prevalent in the poor sleep quality group (10). Although the aforementioned studies included important aspects of the relationship between DR and sleep disturbance, the sample size was too small to represent the clinical population. Moreover, these studies did not account for important DM-related variables, such as DM duration, insulin therapy, hypoglycemic medication, or comorbid DM complications. Controlling for such covariates is imperative to reveal the relationship between sleep disturbances and DR.

In this regard, we aimed to elucidate the relationship between DR and insomnia in patients with type 2 diabetes by considering important demographic factors and DM-related variables and utilizing a nationwide population-based claims database that can effectively represent the real-world setting. We hypothesized that the insomnia risk increases significantly in patients with DR and that insomnia risk is affected by significant interactive associations between DR, demographic factors, and DM-related variables.

## Material and Methods

### Data Source and Study Population

In South Korea, the National Health Insurance System mandates regular health examinations for the public. A biannual health check-up was conducted for every individual registered in the health insurance service. In the present study, subjects aged ≥ 20 years who underwent a health examination between 2009-2012, with prevalent type 2 diabetes regardless of onset, were included. Those with missing health examination parameters or a history of insomnia (those with a history of one or more diagnosis of G470 regardless of outpatient visits or hospitalization status) were excluded. Outcome events were estimated after a lag of one year, and events within one year were excluded. The index represents the date of the last health examination. The study population was followed until a new diagnosis of insomnia, censoring by death, or the end of the study (December 31, 2019).

### Disease Definition and Outcome Variable

Type 2 diabetes was diagnosed based on the following definitions: (i) records of at least one annual claim for an anti-diabetic medication prescription under the International Statistical Classification of Diseases, Tenth Revision (ICD-10) codes E11-14 from the insurance claims database, or (ii) fasting plasma glucose ≥126 mg/dL in the health examination without a history of previous prescription of anti-diabetic medication. Anti-diabetic medications were defined as sulfonylureas, metformin, DPP4 inhibitors, thiazolidinediones, alpha-glucosidase inhibitors, meglitinides, and insulins. Prescribed medications were identified at the index year, and the DM duration was estimated based on the first diagnosis of type 2 diabetes to the index date. Comorbidities were defined using ICD-10 diagnostic codes with regard to the subjects’ healthcare usage, medication prescription, or health examination results (11). Patients with type 2 diabetes were consecutively subdivided into DR, proliferative DR (PDR), and NPDR (non-proliferative diabetic retinopathy) groups. DR was defined as a history of two or more outpatient clinic visits or one or more hospitalizations within a year of the index date, and with a DR diagnostic code of H360 in patients with type 2 diabetes. PDR was defined as a DR diagnosis with the procedural codes S5160 and S5161 for pan-retinal photocoagulation. NPDR was defined as a DR diagnosis without procedural codes S1560 and S5161. Newly diagnosed patients with insomnia were defined as those who had two or more outpatient clinic visits with a diagnostic code of G470 during the follow-up period based on the claims data.

### Baseline Data and Comorbidities

Baseline demographic data were based on the information provided from the first health examination. Every subject went through measurements of height, weight, body mass index (BMI), fasting blood glucose (FBS), total cholesterol, high-density lipoprotein HDL-cholesterol (HDL-C), low-density lipoprotein LDL-cholesterol (LDL-C), estimated glomerular filtration rate (eGFR), and systolic blood pressure (SBP), and diastolic blood pressure (DBP) a t the first health examination. Income level was dichotomized at the lowest 25%. Past health-related behaviors, including smoking, drinking, and physical activity, were collected with self-reported surveys. Detailed classification and criteria of health-related behaviors are summarized in **Supplementary Table 1**. Comorbidities such as hypertension, dyslipidemia, and chronic kidney disease (CKD) were defined using ICD-10 diagnostic codes with regard to the subjects’ health care usage, health examination results, or medication prescription. Detailed comorbidity definitions and descriptions are summarized in **Supplementary Table 2**.

### Statistical Analysis

Baseline characteristics were analyzed with descriptive statistics, which were presented as means with standard deviations or as numbers and percentages. The incidence rates of insomnia in DR, PDR, and NPDR were estimated per 1000 person-years. Cox proportional hazard regression analysis was conducted to explore the risk of insomnia in DR, PDR, and NPDR, with adjustments for the following baseline covariates: age, sex, hypertension, dyslipidemia, smoking, drinking, regular exercise, body mass index, fasting blood glucose level, insulin use, number of anti-diabetic medications, and DM duration. Subgroup analyses were sequentially conducted with stratifications according to age (three strata: <40, 40-64, ≥ 65 years) and sex. Moreover, DM-related variables, DM duration, insulin use, number of anti-diabetic medications, and CKD status, were dichotomized to see their interactive effects with DR and& PDR status on the incidence of insomnia. Statistical significance level was defined as a two-sided P-value less than 0.05. All statistical analyses were performed using SAS software (ver.9.4; SAS Institute, Cary, NC, USA).

### Ethics Statement

The institutional review board of St. Vincent’s Hospital, Suwon, Korea approved the study (No. VC22ZASI0044) and waived the requirement for informed consent since patient data has been deidentified.

## Results

### Demographic Characteristics of the Participants

The baseline demographic and medical characteristics of the study population are summarized in **Table 1**. There were significant differences in age, sex, hypertension, dyslipidemia, smoking, drinking, and regular exercise status between the two groups. As for the DM-related variables, the proportion of the population on insulin or ≥3 oral anti-diabetic medications was different between the DR and Non-DR groups and PDR and NPDR groups. At the first health examination, the BMI, FBS, eGFR, total cholesterol, HDL-C, LDL-C, and DBP were measured. There were significant differences in age, sex, hypertension, dyslipidemia, smoking, drinking, and regular exercise status between the two groups. As for the DM-related variables, the proportion of the population on insulin and ≥3 oral anti-diabetic medications was different between the two groups. At the first health examination, BMI, FBS, eGFR, total cholesterol, HDL-C, LDL-C, SBP, and DBP differed between the two groups.

**Table 1:** Baseline characteristics of the study population.

N	Non-DR	DR	P-value	NPDR	PDR	P-value
	2093687	112932		101371	12686	
**Age, years**	56.13 ± 12.31	62.44 ± 9.71	<.0001	62.78 ± 9.66	59.51 ± 9.69	<.0001
< 40	186837 (8.92)	1465 (1.3)	<.0001	1220 (1.2)	268 (2.11)	<.0001
40 - 64	1357725 (64.85)	61395 (54.36)		53726 (53)	8376 (66.03)	
≥ 65	549125 (26.23)	50072 (44.34)		46425 (45.8)	4042 (31.86)	
**Sex, male**	1334288 (63.73)	57324 (50.76)	<.0001	50678 (49.99)	7301 (57.55)	<.0001
**Income, low 25%**	434550 (20.76)	23475 (20.79)	0.7987	20950 (20.67)	2752 (21.69)	0.7987
**Hypertension, yes**	1134728 (54.2)	79267 (70.19)	<.0001	70779 (69.82)	9286 (73.2)	<.0001
**Dyslipidemia, yes**	831044 (39.69)	60448 (53.53)	<.0001	54328 (53.59)	6693 (52.76)	<.0001
**Smoke**			<.0001			<.0001
Non	1104687 (52.76)	75461 (66.82)		68188 (67.27)	7956 (62.71)	
Ex	402061 (19.2)	21930 (19.42)		19593 (19.33)	2581 (20.35)	
Current	586939 (28.03)	15541 (13.76)		13590 (13.41)	2149 (16.94)	
**Drink**			<.0001			<.0001
Non	1123840 (53.68)	83631 (74.05)		75046 (74.03)	9385 (73.98)	
Mild	741937 (35.44)	23984 (21.24)		21521 (21.23)	2730 (21.52)	
Heavy	227910 (10.89)	5317 (4.71)		4804 (4.74)	571 (4.5)	
**Regular exercise, yes**	430197 (20.55)	28171 (24.95)	<.0001	25420 (25.08)	2981 (23.5)	<.0001
**Insulin, yes**	119473 (5.71)	37165 (32.91)	<.0001	31755 (31.33)	5767 (45.46)	<.0001
**3 ≥ oral** **Anti-diabetic medication, yes**	244538 (11.68)	38148 (33.78)	<.0001	33577 (33.12)	4917 (38.76)	<.0001
**DM duration, years**	2.56 ± 3.2	6.61 ± 2.52	<.0001	6.59 ± 2.53	6.72 ± 2.51	<.0001
≥ 5years	557657 (26.64)	88188 (78.09)	<.0001	78881 (77.81)	10091 (79.54)	<.0001
**At the first health examination**						
**BMI, kg/m^2^**	25.13 ± 3.4	24.49 ± 3.19	<.0001	24.53 ± 3.2	24.15 ± 3.09	<.0001
**FBS., mg/dL**	145.86 ± 46.46	144.77 ± 54.89	<.0001	143.79 ± 53.77	152.89 ± 62.98	<.0001
**eGFR, mL/min/1.73m^2^**	85.98 ± 36.25	77.59 ± 36.47	<.0001	77.9 ± 36.43	75.1 ± 37.31	<.0001
**Total cholesterol, mg/dL**	197.65 ± 42.17	181.82 ± 41.32	<.0001	181.42 ± 40.9	185.18 ± 44.35	<.0001
**HDL-C, mg/dL**	51.91 ± 21.85	50.45 ± 22.7	<.0001	50.56 ± 22.93	49.65 ± 21.83	<.0001
**LDL-C, mg/dL**	111.91 ± 40.85	102.52 ± 38.39	<.0001	102.15 ± 38.19	105.52 ± 40.14	<.0001
**SBP, mmgHg**	129.09 ± 15.78	129.04 ± 16.29	0.2938	128.91 ± 16.14	130.08 ± 17.32	0.2938
**DBP, mmHg**	79.35 ± 10.28	76.69 ± 10.05	<.0001	76.62 ± 9.99	77.33 ± 10.5	<.0001

Values are presented as the mean ± standard deviation or number (%). DR, diabetic retinopathy; NPDR, non-proliferative diabetic retinopathy; PDR, proliferative diabetic retinopathy; DM, diabetes mellitus; BMI, body mass index; FBS, fasting blood glucose; GFR, glomerular filtration rate; HDL-C, high-density lipoprotein cholesterol; LDL-C, low-density lipoprotein cholesterol; SBP, systolic blood pressure; DBP, diastolic blood pressure.

### Incidence Rate and Risk of Insomnia in DR and PDR

The risk of insomnia is shown in **Table 2**. The incidence rates of insomnia were higher in the DR group than in the non-DR group, with incidence rates of 15.521 per 1,000 person-years and 25.8854 per 1,000 person-years, respectively. The risk of insomnia was significantly higher in the DR group than in the non-DR group [adjusted hazard ratio (aHR)=1.306, 95% confidence interval (CI): 1.288-1.325] after controlling for age and sex. After controlling for additional covariates, such as hypertension, dyslipidemia, smoking, drinking, regular exercise status, BMI, FBS, insulin status, number of anti-diabetic medications, and DM duration, the risk remained higher in the DR group (aHR=1.125, 95% CI:1.108-1.142). On analyses of the subgroups of DR, considering PDR status, the PDR group demonstrated a higher risk of insomnia incidence (aHR:1.485, 95% CI:1.425-1.548) when compared the NPDR and non-DR groups, even after controlling for age and sex. When additional confounding factors such as hypertension, dyslipidemia, smoking, drinking, regular exercise status, BMI, FBS, insulin status, number of anti-diabetic medications, and DM duration were controlled for, the PDR group demonstrated the highest risk of insomnia incidence (aHR=1.205, 95% CI: 1.156-1.256).

**Table 2:** Incidence rate (IR) and risk of insomnia according to diabetic retinopathy (DR) and proliferative diabetic retinopathy (PDR) diagnosis.

	N	Insomnia	Duration	IR (per 1,000)	Model 1	Model 2	Model 3
**Non-DR**	2093687	240946	15523891.88	15.521	1 (ref.)	1 (ref.)	1 (ref.)
**DR**	112932	20677	798790.9	25.8854	1.306 (1.288, 1.325)	1.289 (1.271, 1.308)	1.125 (1.108, 1.142)
**Non-DR**	2092562	240758	15515857.38	15.5169	1 (ref.)	1 (ref.)	1 (ref.)
**NPDR**	101371	18575	717873.08	25.875	1.287 (1.268, 1.307)	1.272 (1.253, 1.291)	1.117 (1.099, 1.134)
**PDR**	12686	2290	88952.32	25.7441	1.485 (1.425, 1.548)	1.454 (1.395, 1.515)	1.205 (1.156, 1.256)

IR: incidence rate.

Model 1: Age, Sex.

Model 2: Age, sex, hypertension, dyslipidemia, smoking, drinking, regular exercise status, and body mass index.

Model 3: Age, sex, hypertension, dyslipidemia, smoking, drinking, regular exercise status, body mass index, fasting blood glucose level, insulin status, number of anti-diabetic medications, and diabetes mellitus duration.

### Incidence Rate and Risk of Insomnia in DR and PDR With Consideration for Age and Sex

The incidence rate and risk of insomnia in the DR group were further analyzed considering age and sex. The incidence of insomnia was consistently higher in all three age groups with DR than in those without Non-DR (**Table 3**). In addition, there was a significant interactive effect between age and DR status on the risk of insomnia (p<0.001). The youngest age group, the population aged <40 years, had the highest risk of insomnia (aHR=1.819, 95% CI:1.516-2.183) when compared with the same age group with non-DR. As for the age group 40-64 years, aHR was also higher in the DR group (aHR=1.211, 95% CI:1.186-1.238). In the elderly aged ≥ 65 years, the aHR consistently remained higher in the DR group (aHR=1.055, 95% CI:1.034-1.076). Meanwhile, there was a significant interactive effect between sex and DR status on the risk of insomnia. (p<0.0001) The risk of insomnia incidence was consistently higher in the DR group for both men and women, but the risk was slightly higher among men (aHR=1.253, 95% CI: 1.226-1.28) than among women (aHR 1.032, 95% CI:1.012-1.053). When the severity of DR was considered, there was a significant interactive effect of age and PDR status on the risk of insomnia. The youngest age group with PDR showed the highest risk (aHR=3.575, 95% CI:2.619-4.88). Consistently, there was a significant interactive effect of sex and PDR status on the risk of insomnia. Men with PDR were at a higher risk of insomnia (aHR=1.307, 95% CI:1.235-1.383) when compared with women with PDR. The risk of insomnia increased with DR severity among both men and women.

**Table 3:** Incidence rate (IR) and risk of insomnia in diabetic retinopathy (DR) and proliferative diabetic retinopathy (PDR) with consideration for the interactive effect of age and sex.

	DR & PDR status	N	Insomnia	Duration	IR (per 1,000)	Model 3	P for interaction
**< 40**	Non-DR	186837	6095	1471541.61	4.1419	1 (ref.)	<.0001
	DR	1465	118	11509.91	10.252	1.819 (1.516, 2.183)	
**40 - 64**	Non-DR	1357725	132379	10337623	12.8056	1 (ref.)	
	DR	61395	9636	454573.88	21.1979	1.211 (1.186, 1.238)	
**≥ 65**	Non-DR	549125	102472	3714727.28	27.5853	1 (ref.)	
	DR	50072	10923	332707.1	32.8307	1.055 (1.034, 1.076)	
**Male**	Non-DR	1334288	127503	9956733.24	12.8057	1 (ref.)	<.0001
	DR	57324	9613	402978.01	23.8549	1.253 (1.226, 1.28)	
**Female**	Non-DR	759399	113443	5567158.65	20.3772	1 (ref.)	
	DR	55608	11064	395812.89	27.9526	1.032 (1.012, 1.053)	
**< 40**	Non-DR	186814	6089	1471388.6	4.1383	1 (ref.)	<.0001
	NPDR	1220	84	9687.35	8.6711	1.544 (1.245, 1.915)	
	PDR	268	40	1975.56	20.2474	3.575 (2.619, 4.88)	
**40 - 64**	Non-DR	1357018	132274	10332459.51	12.8018	1 (ref.)	
	NPDR	53726	8329	399360.6	20.8558	1.195 (1.168, 1.223)	
	PDR	8376	1412	60376.78	23.3865	1.323 (1.255, 1.395)	
**≥ 65**	Non-DR	548730	102395	3712009.27	27.5848	1 (ref.)	
	NPDR	46425	10162	308825.13	32.9054	1.058 (1.036, 1.08)	
	PDR	4042	838	26599.98	31.5038	1.015 (0.948, 1.087)	
**Male**	Non-DR	1333633	127404	9952083.89	12.8017	1 (ref.)	<.0001
	NPDR	50678	8489	356675.57	23.8003	1.245 (1.217, 1.274)	
	PDR	7301	1223	50951.78	24.0031	1.307 (1.235, 1.383)	
**Female**	Non-DR	758929	113354	5563773.49	20.3736	1 (ref.)	
	NPDR	50693	10086	361197.5	27.9238	1.025 (1.004, 1.047)	
	PDR	5385	1067	38000.54	28.0785	1.106 (1.041, 1.175)	

Model 3: Age, sex, hypertension, dyslipidemia, smoking, drinking, regular exercise status, body mass index, fasting blood glucose level, insulin status, number of antidiabetic medications, and diabetes mellitus duration.

### Incidence Rate and Risk of Insomnia in DR and PDR With Consideration for DM-Related Variables

When DM-related variables were considered, a significant interactive effect of DM duration was observed. Effect of CKD status and DR status on insomnia incidence is presented in **Table 4**. Regardless of DM duration (less than 5 years or ≥ 5 years of DM diagnosis), the DR group was exposed to a higher risk of insomnia than the non-DR group. The risk was slightly higher in the DM duration of <5 years’ group (aHR=1.171, 95% CI:1.134-1.209) when compared with the group with a DM duration of ≥5 years (aHR=1.113, 95% CI:1.095-1.132). The risk was consistently higher in the DR group, regardless of the insulin status or number of oral anti-diabetic medications. The CKD status significantly affected the risk of insomnia in the DR group, with a higher risk of insomnia observed in the DR group with CKD (aHR=1.233, 95% CI:1.199-1.268) than in the DR group without CKD (aHR=1.084, 95% CI:1.065-1.103).

**Table 4:** Incidence rate (IR) and risk of insomnia in diabetic retinopathy (DR) and proliferative diabetic retinopathy (PDR) with consideration for the interactive effect of diabetes mellitus (DM)-related variables.

	DR & PDR status	N	Insomnia	Duration	IR (per 1,000)	Model 3	P for interaction
**DM duration < 5years**	Non-DR	1536030	157096	11472011	13.6938	1 (ref.)	0.0055
	DR	24744	3942	181251.6	21.7488	1.171 (1.134, 1.209)	
**DM duration ≥ 5years**	Non-DR	557657	83850	4051881	20.6941	1 (ref.)	
	DR	88188	16735	617539.3	27.0995	1.113 (1.095, 1.132)	
**Insulin** (–)	Non-DR	1974214	219906	14700848	14.9587	1 (ref.)	0.7568
	DR	75767	12824	549886.2	23.3212	1.123 (1.103, 1.144)	
**Insulin (+)**	Non-DR	119473	21040	823043.8	25.5636	1 (ref.)	
	DR	37165	7853	248904.7	31.5502	1.129 (1.1, 1.159)	
**3 < oral** **anti-diabetic**	Non-DR	1849149	204307	13728043	14.8825	1 (ref.)	0.1119
**medication**	DR	74784	13478	529329.8	25.4624	1.135 (1.114, 1.156)	
**3 ≥ oral** **anti-diabetic**	Non-DR	244538	36639	1795849	20.4021	1 (ref.)	
**medication**	DR	38148	7199	269461.1	26.7163	1.107 (1.079, 1.135)	
**CKD** (–)	Non-DR	1887666	206590	14088198	14.664	1 (ref.)	<.0001
	DR	86990	14727	631592	23.3173	1.084 (1.065, 1.103)	
**CKD (+)**	Non-DR	206021	34356	1435694	23.9299	1 (ref.)	
	DR	25942	5950	167198.9	35.5864	1.233 (1.199, 1.268)	
**DM duration < 5years**	Non-DR	1535689	157051	11469521	13.6929	1 (ref.)	0.0026
	NPDR	22490	3636	164882.4	22.0521	1.173 (1.135, 1.213)	
	PDR	2595	351	18859.47	18.6113	1.145 (1.031, 1.272)	
**DM duration ≥ 5years**	Non-DR	556873	83707	4046336	20.6871	1 (ref.)	
	NPDR	78881	14939	552990.7	27.0149	1.102 (1.083, 1.122)	
	PDR	10091	1939	70092.86	27.6633	1.214 (1.161, 1.271)	
**Insulin (-)**	Non-DR	1973446	219794	14695220	14.9568	1 (ref.)	0.0026
	NPDR	69616	11898	505173.9	23.5523	1.124 (1.102, 1.145)	
	PDR	6919	1038	50340.49	20.6196	1.114 (1.048, 1.185)	
**Insulin (+)**	Non-DR	119116	20964	820637.4	25.546	1 (ref.)	
	NPDR	31755	6677	212699.2	31.3917	1.105 (1.075, 1.136)	
	PDR	5767	1252	38611.84	32.4253	1.293 (1.221, 1.369)	
**3 < oral** **anti-diabetic medication**	Non-DR	1848370	204172	13722513	14.8786	1 (ref.)	0.1615
	NPDR	67794	12219	480517	25.4289	1.127 (1.105, 1.148)	
	PDR	7769	1394	54343.47	25.6517	1.228 (1.164, 1.295)	
**3 ≥ oral** **anti-diabetic medication**	Non-DR	244192	36586	1793345	20.401	1 (ref.)	
	NPDR	33577	6356	237356.1	26.7783	1.097 (1.068, 1.127)	
	PDR	4917	896	34608.86	25.8893	1.17 (1.095, 1.251)	
**CKD (-)**	Non-DR	1886803	206466	14081906	14.6618	1 (ref.)	<.0001
	NPDR	78603	13379	570850.6	23.437	1.082 (1.062, 1.102)	
	PDR	9250	1472	67033.02	21.9593	1.095 (1.04, 1.153)	
**CKD (+)**	Non-DR	205759	34292	1433951	23.9143	1 (ref.)	
	NPDR	22768	5196	147022.5	35.3415	1.208 (1.173, 1.245)	
	PDR	3436	818	21919.3	37.3187	1.448 (1.351, 1.553)	

Model 3: Age, sex, hypertension, dyslipidemia, smoking, drinking, regular exercise status, body mass index, fasting blood glucose level, insulin status, number of antidiabetic medications, and DM duration. CKD, chronic kidney disease.

When the PDR status was considered, DM duration, insulin status, and CKD status all displayed significant interactive effects with PDR stage on the risk of insomnia (**Table 4**). In the PDR group, those with a DM duration ≥5 years had the highest risk of insomnia (aHR=1.214, 95% CI:1.161-1.271). Insulin status also significantly affected the risk, with the highest risk of insomnia observed in the PDR group on insulin (aHR=1.293, 95% CI=1.221-1.369). Regardless of the number of oral anti-diabetic medications, the PDR group consistently exhibited the highest risk of insomnia compared with the NPDR or non-DR groups. As with the previous results on the comparison between the DR and non-DR groups, the PDR group with CKD was vulnerable to the risk of insomnia (aHR: 1.448, 95% CI:1.351-1.553), while a slightly less increased risk was observed in NPDR with CKD (aHR:1.208, 95% CI:1.351-1.553).

## Discussion

To the best of our knowledge, this is the first study to demonstrate the relationship between DR and insomnia by analyzing large cohort from a health insurance database, with stringent classification of DR subjects and control for demographic and DM-related variables. The major findings of the current study are as follows: 1) insomnia risk was significantly increased in the DR group, and a higher risk was noted in the PDR group; 2) males and the youngest age group with DR were more vulnerable to insomnia; 3) there was an interactive effect of DR status and DM duration on increasing insomnia risk; and 4) there was an interactive effect of PDR status and DM duration, insulin therapy status, and CKD status on increasing insomnia risk.

### Demographic Characteristics of DR and PDR

Demographic characteristic results observed in our study are in line with previous studies where a close association between macrovascular and microvascular complications was consistently replicated, with increased cerebrovascular events in DR when compared with non-DR (12). Indeed, the DR group in our study population demonstrated worse metabolic profiles than their counterparts. In the comparison between the PDR and NPDR groups, worse metabolic profiles were noted in the PDR group. According to a previous systematic review and meta-analysis, those with PDR or diabetic macular edema were more likely to be exposed to an increased risk of new cardiovascular events (13), and when compared with NPDR, PDR was associated with a higher risk of cardiovascular diseases (14). In addition, advanced-stage DR is associated with underlying subclinical cardiovascular diseases (15).

The longer DM duration, high proportion of insulin therapy, and multiple anti-diabetic medications noted in the DR and PDR groups are concordant with a previous study where the risk of DR was increased in DM patients with longer illness duration, poor glycemic control, and high SBP (16). Moreover, the decreased GFR in the DR and PDR groups is also in line with a previous result, where DR was proposed as a harbinger of future CKD in patients with DM (17). The demographic characteristics of our study population highlight the close interaction between microvascular and macrovascular complications in DM patients.

### Increased Insomnia Risk in DR an PDR

The incidence of insomnia was significantly higher in the DR group than in the non-DR group. Moreover, the risk of insomnia was higher in the DR group than in the non-DR group, and the risk increased proportionally with the severity of DR. The reason for the increased insomnia risk may be attributable to circadian disruption induced by DR, since the retina is a receptor of light source indispensable for sleep-wake circadian rhythm. A recent study on patients with DR reported that fewer photosensitive ganglion cells and a reduction in retinal function were associated with lower urinary melatonin levels, which may result in circadian disruption and consequent sleep disturbance (18). Moreover, DR significantly disrupted the expression of melanopsin-expressing retinal ganglion cells, which are critical for the regulation of melatonin levels and circadian rhythms (19). Indeed, melatonin levels were significantly reduced in DR patients in a recent study (20), and dim-light onset melatonin was significantly earlier in DR group than the control group (21). Circadian rhythm disruption is often delineated as a culprit for progression of insomnia (22), and retinal dysfunction in DR may have accelerated the aforementioned process. Moreover, insomnia in DR may be a manifestation of circadian disruption in DR patients, since many of patients with circadian dysfunction present with complaints of insomnia (23). The proportional increase in insomnia risk with DR severity further supports our proposition that increases in retinal dysfunction and circadian rhythm disruption are closely related to insomnia risk.

Recent research has highlighted the importance of the retinal circadian clock in promoting photoreceptor health (21), and how circadian rhythm dysregulation can instigate and aggravate DR pathology (24). In this respect, circadian disruption manifesting as insomnia warrants close clinical attention. Drugs targeting circadian rhythm dysfunction are being investigated (25), and future studies on the impact of these drugs on insomnia and circadian rhythm in DR patients will further expand our knowledge of DR pathogenesis and prevention.

### Impact of Age and Sex on the Insomnia Risk in DR and PDR

According to our results, the youngest age group (<40 years) and male DR patients were more vulnerable to insomnia. Moreover, age and sex had a significant interactive effect with DR and PDR status in increasing the insomnia risk. Several interpretations can be inferred from these results. Regarding male vulnerability to insomnia, disparities in sex-related hormonal influences on the retina and circadian rhythm should be considered. Although controversies exist, estrogen has been reported to play a disparate role according to different stages of DR and is protective in milder forms of DR by initiating repair processes (26). Moreover, a recent study demonstrated that microvascular damage, represented by retinal arteriole and venule diameters, is associated with increased androgenic profiles (27). As for circadian endocrine rhythms, the retinohypothalamic tract, one of the major afferent pathways of the suprachiasmatic nucleus, expresses estrogen or androgen receptors (28). Moreover, both estrogen and androgen receptors are expressed in the human retina (28). Sex disparities in insomnia risk in DR and PDR may be attributed to the aforementioned evidence. Additionally, considering that insomnia prevalence in the general population is higher in females (29), male vulnerability to insomnia in DR and PDR population may represent different underlying mechanisms involved in the pathogenesis of insomnia in DR and PDR. Further studies are needed to clarify sexual dimorphism in the association between insomnia and DR.

Meanwhile, the increased risk of insomnia in the youngest DR and PDR groups (three-fold increase in the youngest PDR group) warrants special clinical attention. In a recent Korean epidemiological study, insomnia was found to be more common among the elderly (30). However, in the present DR and PDR study population, the insomnia incidence was higher in the youngest age group. Emerging evidence indicates that young-onset type 2 diabetes patients are intrinsically more susceptible to DR (31) and that young adults are more vulnerable to repeated circadian disruption and sleep deprivation than the elderly (32). Further, young adults were more prone to skip DR screening attendance, which meant that there were many hurdles in the focused management of DR in this group (33). Our results are also in line with a previous study according to which the chronic course and complications of DM can negatively impact young adults’ mental health and increase stress, since young adults have relatively more years of life expectancy (34). Poor management of DR, stress, and inherent vulnerability to circadian disruption may have increased the insomnia risk in this group.

### Impact of DM Duration, Insulin Therapy and CKD Status on the Insomnia Risk in DR and PDR

DM duration and DR status had an interactive effect on the risk of insomnia. A higher risk was observed in the DR group with a DM duration <5 years than in the non-DR group, while the highest insomnia risk was observed in the PDR group with a DM duration <5 years. The increased insomnia risk in the DR group with a shorter illness duration may be attributable to the influence of acute stress induced by major DM complications. A short DM duration means that patients are likely to adapt to lifestyle changes and glycemic control. Major complications, such as DR, can inflict a huge psychological burden, resulting in stress and anxiety, which may cause insomnia. While psychological interventions are important in patients with DR (35), studies on crisis interventions for those who have to confront the diagnosis of DR with a relatively shorter DM duration are scarce.

In the PDR group, a higher insomnia risk was observed in the group with a DM duration >5 years, insulin therapy group, and CKD group when compared with their counterparts. Patients with PDR, the most severe form of DR, may be at the greatest risk for retinal damage and resultant circadian disruption, as described in the preceding paragraphs. Moreover, in the PDR group, a longer DM duration and insulin therapy implied that the subjects were exposed to high blood glucose levels and poor glycemic control over a long period of time. Glycosylated hemoglobin was negatively correlated with sleep efficiency in a recent study, although the illness duration of the participants was relatively shorter than that of our study population (36). To date, no confirmatory findings have been reported regarding the relationship between glycemic control and sleep (37). As for the increased risk of insomnia observed in PDR with CKD, the results are in line with those of previous studies. The prevalence of insomnia was as high as 70% in hemodialysis patients with diabetes (38). One study on elderly patients with type 2 diabetes indicated that a longer DM duration, lower eGFR, and presence of nephropathy were all related to increased use of hypnotics (39). Increased insomnia in PDR with CKD may be due to exposure to a chronic inflammatory status and the heavy psychological burden of comorbid conditions (40).

This study has several limitations that must be considered. First, the diagnoses of type 2 diabetes, DR, and PDR were solely based on the ICD-10 codes of the claims data. Second, the depression and anxiety status of the study population were not accounted for, which may have confounded our results. Third, the study may not have allowed sufficient follow-up time to accurately represent the actual incidence of insomnia in DR. Despite the aforementioned limitations, this was the first attempt to examine the relationship between insomnia incidence in DR and utilization of big claims databases. We discovered a significant increase in insomnia risk in the DR and PDR groups and an intricate interplay between DR, PDR status, demographic factors, and DM-related variables in increasing insomnia risk. Further studies are required to confirm this relationship.

## Data Availability Statement

The datasets presented in this article are not readily available because the database is only accessible to the investigators who have been approved to utilize the health claims database of Korea. Requests to access the datasets should be directed to https://opendata.hira.or.kr/home.do.

## Ethics Statement

The studies involving human participants were reviewed and approved by the institutional review board of of St. Vincent’s Hospital, Suwon, Korea approved the study (No. VC22ZASI0044) and waived the requirement for informed consent since patient data has been deidentified. Written informed consent for participation was not required for this study in accordance with the national legislation and the institutional requirements.

## Author Contributions

YHU, data interpretation, drafting the article, design of the work,and critical revision of the article; T-WK, J-HJ, and S-CH, critical revision of the article and final approval of the version to be published; H-JS, design of the work, critical revision of the article, and final approval of the version to be published K-DH, analysis of data, critical revision of the article, and final approval of the version to be published. All authors contributed to the article and approved the submitted version.

## Funding

The authors wish to acknowledge the financial support of Clinical Research Invigoration Project of the St. Vincent’s Hospital, The Catholic University of Korea (IRB No: VC22ZASI0044). Also, this work was supported by the National Research Foundation of Korea(NRF) grant funded by the Korea government (MSIT) (No. 2020R1I1A1A01057792).

## Conflict of Interest

The authors declare that the research was conducted in the absence of any commercial or financial relationships that could be construed as a potential conflict of interest.

## Publisher’s Note

All claims expressed in this article are solely those of the authors and do not necessarily represent those of their affiliated organizations, or those of the publisher, the editors and the reviewers. Any product that may be evaluated in this article, or claim that may be made by its manufacturer, is not guaranteed or endorsed by the publisher.
