# Supplementary material for: Association Between Diabetic Retinopathy and Insomnia Risk: A Nationwide Population-Based Study
Source: Front Endocrinol (Lausanne). 2022 Jul 14;13:939251. doi: 10.3389/fendo.2022.939251 (PMC9333090; doi:10.3389/fendo.2022.939251)
Supplement: Supplementary file 1 [file Table_1.docx]

**Supplementary Table 1. Definitions of comorbidities**

|  | ICD-10 codes with additional definitions | | Health examinations results |
| --- | --- | --- | --- |
| Comorbidities |  |  |  |
| Hypertension | I10-I13, I15 | Admission or outpatient department≥1, and prescription of anti-hypertensive drug (thiazide, loop diuretics, aldosterone antagonist, alpha-/beta-blocker, calcium-channel blocker, angiotensin-converting enzyme inhibitor, angiotensin II receptor blocker) | Systolic blood pressure ≥140 mmHg or diastolic blood pressure ≥90 mmHg |
| Dyslipidemia | E78 | Admission or outpatient department≥1, and prescription of lipid-lowering medication (statin, ezetimibe, fenofibrate) | Fasting total cholesterol ≥240 mg/dL |
| Chronic kindey disease |  |  | Glomerular filtration rate<60mL/min/1.73m^2^ |

**Supplementary Table 2. Definitions of health-related behaviors.**

|  | Category | Definition |
| --- | --- | --- |
| Smoking status | Non-smoker | Never-smoker or lifetime smoking history of <5 packs (100 cigarettes) |
|  | Ex-smoker | Lifetime smoking history of ≥5 packs (100 cigarettes) and quit smoking |
|  | Current smoker | Lifetime smoking history ≥5 packs (100 cigarettes) and currently smoking |
| Drinking status | Non-drinker | Daily alcohol intake = 0 |
|  | Mild drinker | Alcohol intake <30g/day (and more than 0) |
|  | Heavy drinker | Alcohol intake ≥30g/day |
| Regular exercise |  | At least 150 minutes per week of moderate-intensity exercise or at least 60 minutes per week of vigorous-intensity exercise   - Moderate-intensity exercise: Exercise requiring a little more exertion or causing a little more breathlessness than usual - Vigorous-intensity exercise: Exercise requiring much more exertion or causing much more breathlessness than usual |
